# A natural experiment to assess recess frequency on children’s physical activity in Arizona (U.S.) elementary schools

**DOI:** 10.1186/s12889-023-17605-4

**Published:** 2024-01-18

**Authors:** Allison Poulos, Kylie Wilson, Marissa Schulke, Kahyun Nam, Punam Ohri-Vachaspati, Yang Bai, Pamela Hodges Kulinna

**Affiliations:** 1 College of Health Solutions, Arizona State University, ABC 222 425 North 5th Street, Phoenix, AZ 85004 USA; 2 Mary Lou Fulton Teachers College, Arizona State University, Tempe, AZ 85281 USA; 3 Department of Health and Kinesiology, University of Utah, Salt Lake City, UT 84112 USA

**Keywords:** Recess dose, Accelerometer, Direct observation, Movement, Policy

## Abstract

**Background:**

In the United States, the number of state policies mandating recess in schools has rapidly increased over the past decade; however, few policies specify recess frequency. Informed by an ecological model of physical activity (PA) policy, this study examined and compared total amounts and intensity of PA expended during recess among children attending schools in compliance with Arizona recess policy ARS§ 15–118 mandating 2 + daily recess periods versus not.

**Methods:**

PA during recess was measured among grade three children (ages 8–10) in four randomly selected elementary schools (two complying averaging 30 daily recess minutes; two non-complying averaging 15 daily recess minutes) in Maricopa County, Arizona. Group-level PA was assessed by direct observation using the System for Observing Play and Leisure (137 observations). A subset of students (N = 134) from all schools wore ActiGraph GT3X + devices during recess to measure individual PA. General linear mixed effects models were used to analyze the impact of recess frequency on group and individual PA during recess.

**Results:**

Students attending complying schools spent significantly greater proportions of time in moderate-to-vigorous PA (MVPA) based on direct observation (5%) and accelerometry (15%) and less time being sedentary based on accelerometry (14%) during recess. Across the school day, this would equate to 5.1 more MVPA minutes based on systematic direct observation and 9.5 more MVPA minutes based on accelerometry, and 4.1 less minutes being sedentary based on accelerometry if students received two daily 15-minute recess periods compared to one.

**Conclusions:**

Students attending elementary schools implementing 2 + recesses, in accordance with state policy, demonstrated greater MVPA and less sedentary time, providing preliminary evidence that recess frequency is associated with greater PA intensity among children during recess. Schools that adhere to state-level PA policies may provide a more supportive environment for PA, resulting in increased movement among students. Specifying recess frequency should be considered in statewide recess policy.

**Supplementary Information:**

The online version contains supplementary material available at 10.1186/s12889-023-17605-4.

## Background

Obesity rates among children in the United States (US) have tripled in the past three decades [1], contributing to increased risks of cardiovascular disease [2], type 2 diabetes [3], and mental health problems such as anxiety and depression [4]. Insufficient physical activity (PA) and increased sedentary behavior are associated with excess weight gain [5] and obesity [2] among children. Urgent action is needed to address high levels of inactivity [6] as more than 75% of US youth do not meet the recommended daily 60 min of moderate-to-vigorous PA (MVPA) [7, 8] and poor mental health among US youth is increasing [9]. Additionally, sedentary time among children is on the rise nationwide [10], further contributing to higher risk of weight gain [11], obesity-related health issues such as cardiometabolic diseases and certain cancers [12], and poor mental health [13].

Schools are a critical source of daily PA due to their accessibility, structure, and systems to support healthy behaviors and health behavior change [14]. Schools are the only setting that reaches nearly all children in the US [15–18], and most children spend approximately half of their waking hours at school [19]. School-based PA is positively related to children’s cognitive functioning [20] and the mental health of children and adolescents [21]. In elementary schools, physical education, recess, classroom-based PA, and other before- and after-school programs contribute substantially to MVPA accumulation [22–25]. Among these, recess stands out as the most significant source of PA at school, accounting for up to 44% of all PA during school hours [26] and helping to counter sedentary time [27, 28].

Although the majority of elementary schools in the US (83%) provide students with one daily recess period [29, 30], less than one quarter (21%) provide recess twice a day [31]. In 2019, Arizona (AZ) enacted a statewide law (ARS§ 15–118) requiring all K-5 schools to offer at least two daily recess periods. This legislation represents one of the first state-level policies in the US specifically addressing *recess frequency*. According to the law, “Each school district and charter school shall provide at least two recess periods during the school day for pupils in kindergarten programs and grades 1 through 5 … a school district or a charter school may count a pupil’s participation in a physical education course during a day as one of that day’s recess periods…” [32]. Duration is not specified in the AZ state law.

Increasing recess frequency offers a cost-effective, accessible, and sustainable opportunity to improve children’s health on a population level and school-based PA policy is widely recognized as one solution to combat global physical inactivity, as a generally small per-capita expense with broad reach [33]. However, there is limited research linking school policy to PA outcomes, and the specific effect of recess frequency on PA remains unknown. There is a need to examine children’s PA in scenarios that differ in recess frequency as some research has suggested a *compensation effect* where one school PA opportunity replaces another (e.g., increased PA at recess may be associated with decreased PA during physical education) [34]. Additionally, most studies on school-based PA policies rely on school staff-reported measures [35], rather than objective assessments [36, 37]. Understanding the optimal frequency of recess can guide consistent policy recommendations, given the significant variation in current recess legislation and practices [38].

This cross-sectional natural experiment is part of a larger longitudinal investigation of PA policies and practices in AZ elementary schools. The larger project is framed on an adapted ecological model of PA policy [19] to assess the implementation and dissemination stages of ARS§ 15–118 affecting children’s PA (see model in Appendix 1). The purpose of this study was to gather preliminary results on the impact of recess frequency on individual and group measured PA, including MVPA, light physical activity (LPA), and sedentary behavior (SB) during recess. Given the existing literature reporting differences in PA levels between boys and girls [39, 40], differences in both individual and group PA between sexes were also assessed.

## Methods

### Study design

This study used a cross-sectional quasi-experimental design to compare the impact of recess frequency on objectively measured individual and group-level PA between students attending schools in compliance with recess policy ARS§ 15–118 (2 + daily recesses) and those not (< 2 daily recesses). Four elementary schools were randomly selected to represent policy compliance and non-compliance based on data from a larger longitudinal survey that assessed reported PA policies and practices throughout Arizona [41]. The survey, administered online to school administrators and teachers each fall, collected information on school-, district-, and state-level policies, as well as PA practices among third-grade students (representing the average elementary grade). Further details on the survey and administration can be found in other publications [41, 42].

### Participants and recruitment

Of the 171 schools that responded to the fall 2021 survey conducted among administrators and teachers, 60% offered two or more recesses, while 14% offered less than two recesses for grade three students. Approximately 26% of respondents did not know or did not respond to the question regarding recess frequency. For the current study, two complying and non-complying schools were randomly selected from each category using a random number generator function in Microsoft Excel. Schools were eligible if they: [1] met one of the two frequency categories [2], were a public school located within Maricopa County, the largest county in AZ, where 62% of the state population resides [3, 43], and served K-5 (including K-6/8/12) students. The research team contacted schools through phone calls and emails, providing information about the study and inviting them to participate. Principals who agreed to participate were offered a $50 gift card as an incentive for assisting with recruitment of third grade teachers and students. All third-grade children at participating schools were observed during recess time and invited to wear accelerometers. Children who returned signed parental consent (40% response rate) participated in device-based data collection with accelerometers during recess time. Third-grade teachers received a $50 gift card for help coordinating data collection. Third-grade students who wore an accelerometer during recess received a $10 gift card. Ethical approval was granted from Arizona State University, and written parent consent along with child assent were obtained.

### Data collection

Members of the research team visited third grade classrooms of the participating schools to confirm school schedules with teachers, place devices on child participants, and conduct observational assessments of one or both recess sessions. To mitigate the impact of seasonal variations, data collection took place during warm months (April, May, October 2022) with daily temperatures similar across time points and conducive to outdoor play (average 86 °F/30°C).

**Group-Level Physical Activity**. Direct observations to measure mean group-levels of PA during recess were conducted using the System for Observing Play and Leisure Activity in Youth (SOPLAY) [44]. SOPLAY is a validated systematic observation tool used extensively to measure group-levels of PA during recess and provides for the additional collection of contextual environmental supports such as temperature, presence of organized activity, and equipment. Direct observation of contextual variables produces objective information with strong internal (face) validity [45] that have been shown to affect movement at recess [46–52]. A minimum of two observers collected observations at each school site over three to four school days. Observers were trained using established training protocols that include using standardized online videos and field-based practice until an acceptable reliability of > 80% was met [44]. Maps of each school playground were obtained using aerial images from Google Maps. Playgrounds were divided into smaller target zones for observation. Prior to data collection, observers visited each playground to verify the map and target zones and establish an observation rotation schedule.

During observations, observers systematically recorded environmental supports during recess and categorized students as engaging in vigorous (representing moderate-to-vigorous; MVPA), walking (representing light physical activity; LPA), or sedentary behavior (SB) using established procedures and momentary time sampling techniques [44]. Data were recorded on paper SOPLAY coding sheets attached to clipboards with three-unit counters. Observers conducted visual scans from the left to right, pausing for one second for each child, and tallied activity levels by pressing down on the counters. Separate sweeps were conducted for girls and boys as differences in PA and SB at recess exist between sex, with boys generally engaged in more MVPA compared to girls [53]. Observers systematically rotated throughout established target zones to record activity throughout the entire play area.

*Individual Physical Activity*. A sample of third grade students at the four schools wore ActiGraph GT3X + accelerometer devices (100 Hz; ActiGraph LLC, Pensacola, US), the most commonly used accelerometers in PA research [54], on their dominant wrist for a minimum of five school days. The wrist location was selected to promote greater wear compliance compared to the hip among children [55]. The research team visited each participating classroom to instruct children and teachers on how to wear the devices. Classroom teachers were given an instructional FAQ and asked to record recess times and relevant information (e.g., student absences) on a daily report. Child sex was recorded by either researchers or classroom teachers. Participants received their device to wear from their teachers upon classroom arrival and removed them at the end of each school day.

### Data analysis

Descriptive statistics (means and standard deviations) were conducted to describe the measures of central tendency and dispersion of observational and device-based PA. Proportions of MVPA, LPA, SB were converted to minutes based on a 15-minute recess period, the most common recess length among our sample. Recess at one school (school 4) was 20 min, so PA amounts were adjusted to represent a 15-minute time block for comparison.

**Group-Level Physical Activity**. Mean observation counts derived from direct observation of all third-grade students at participating schools for each PA level were averaged across days and by sex.

**Individual Physical Activity**. We followed recommendations of the National Cancer Institute to promote consistent and standard reporting for accelerometer studies [56], except for minimum wear time since 24-hour measurements were not collected as this study focused only on movement during recess. Choi cut points were used to determine a standard wear time recommended in accelerometer studies with youth [57]. Students with at least one recess of valid wear time were included in the analyses, and non-wear times were excluded from analyses. Recess segments were defined based on school bell schedules. Crouter linear regression vector magnitude cut-points for youth [58] were used to determine levels of PA overall, by school, by gender, and by recess frequency. The device data was processed using ActiLife software (v6.13.4).

**Covariates**. The presence of environmental supports for PA (e.g., presence of equipment, organized activity, temperature) collected during observations as well as school-level income and child sex were controlled for in statistical analyses. Presence of equipment and organized activity (binary) were averaged to produce a scalar variable ranging from 0 (never) to 1 (always). Income was a continuous variable based on the percentage of students eligible for free and reduced-price lunch using 2021–2022 data from the National Center for Education Statistics [59].

### Data analysis

Because student participants were nested within schools, and school settings influence health behaviors [60, 61], multilevel modeling was used to assess the impact of recess frequency on movement during recess (MVPA, LPA, and SB). To examine mean group movement assessed by direct observation and individual movement assessed by accelerometry, general linear mixed effects models (GLMM) with Bonferroni post hoc comparisons were utilized to test the association between recess frequency, taking into account the clustering of students within the same school for the individual data. Separate GLMMs (MVPA, LPA, SB) with covariates described below were fit to both the group and individual PA data.

In the GLMMs examining group PA (SOPLAY observations), recess frequency, sex and the interaction between frequency and sex were also entered as main predictors with school income, organized activity, presence of equipment, and temperature as covariates. For models examining individual PA (accelerometry), recess frequency, sex, and the interaction between frequency and sex were entered as primary predictors with income included as a covariate. Data from students at schools with 2 + recesses were averaged prior to running the GLMMs. Analyses were also conducted without averaging (i.e., two data points for these students); however, we found no changes in statistical outcomes. All models were estimated using a maximum likelihood estimator and significance level of 0.05 using SPSS Statistics for Windows, version 26 (IBM Corp.) [62].

## Results

The average enrollment of the four schools was 739 students, with an approximately even proportion of boys (50%) and girls (50%). Participating schools averaged 86 third grade students, comparable to the average Maricopa County school enrollment of 99 students in grade three [63]. An average of 36 devices were worn per school (142 total, representing 42% of third grade students at all schools). School-level income varied with two schools classified as low-poverty, one as mid-low poverty, and one considered high poverty (Table 1).

Recess length at the non-complying schools was 15 or 20 min, and 15 min at complying schools. On average, children at non-complying schools spent 17.5 daily minutes at recess and children at complying schools spent 30 daily minutes at recess. Equipment was present during recess less than half of the time during observations (46%), while the frequency of organized activities were generally low (17%) across schools during observations. Descriptive information about participating schools and their recess environments are shown in Table 1.

**Table 1:** School-level demographic information and recess characteristics

School	*School-Level Information*						
		Enrollment: Total (#)	Enrollment: Boys (%)	Enrollment: Girls (%)	Enrollment: 3rd Grade	# Devices Worn	Income: FRPM eligibility (%)
Average	-	739.0(251.3)	49.5(2.9)	50.5(2.9)	85.5(24.7)	35.5(4.9)	39.7(31.8)
1 2 3 4	K-8	1024	46.6	53.4	118	35	24.0
	PK-6	570	51.6	48.4	81	42	19.1
	PK-6	490	47.8	52.2	58	35	28.6
	PK-8	872	52.6	47.4	85	30	86.9
School	*Recess Environment*						
	# Recesses Per Day	# Recesses Observed	# SOPLAY Observations^1^	# Target Areas	Equipment (%)^2^	Organized Activity (%)^2^	Temperature (F°)
Average 1 2 3 4	-	22	137	-	45.9(27.8)	17.0(23.9)	86.0
	1	4	24	5	29.1	7.9	86.0
	2	8	50	4	47.6	22.6	81.6
	2	7	51	4	58.8	11.5	91.3
	1	3	12	7	42.0	20.0	74.0

*Note*: FRPM = Free-or-reduced priced meals where ≤ 25% indicates low poverty; 25.1–50% indicates mid-low poverty; 50.1–75% indicates mid-high poverty; >75% indicates high poverty [64]. ^1^Represents observations of complete playground (total scans were divided by the number of target areas at each playground) ^2^Item on a scale where 0 = “Never” to 1 = “Always”, converted to percent

### Group-level physical activity

A total of 137 SOPLAY scans were conducted over 22 recess periods at the four schools. Descriptive results indicated that, on average across schools, most time at recess was spent in LPA (*M* = 43%, *SD* = 7%). When examined by policy compliance category, high frequency schools averaged greater MVPA (29% v. 24%). When converted to minutes (per 15 min), students at high frequency schools engaged in 4.39 min compared to low frequency schools where students engaged in 3.6 min of MVPA per recess. This equates to 8.8 MVPA minutes per day, or an additional 5.1 min per day of MVPA among students attending schools offering two recess periods compared to one. When multiplied by the average 180 days of instruction in U.S. public schools, this equates to 15 h across the school year. Full results by recess frequency for observational data are shown in Table 2. Results of direct observations at individual schools can be found in Appendix 2.

**Table 2:** Mean Recess Physical Activity Levels by Recess Frequency: Direct Observation

Recess Frequency	Sedentary		Light Physical Activity		Moderate-to-Vigorous Physical Activity	
	% *M(SD)*	Minutes *M(SD)*	% (*SD*)	Minutes *M(SD)*	% *M(SD)*	Minutes *M(SD)*
Low (*n* = 2)	30.0(9.7)	4.5(1.5)	45.7(4.0)	6.85(0.61)	24.3(9.6)	3.6(1.4)
Boys	22.9(4.0)	3.4(0.6)	45.7(4.4)	6.85(0.66)	31.5(4.3)	4.7(0.6)
Girls	37.2(8.2)	5.6(1.2)	45.7(4.0)	6.86(0.60)	17.1(7.6)	2.6(1.1)
High (*n* = 2)	28.9(9.2)	4.3(1.4)	41.8(8.0)	6.27(1.20)	29.3(6.3)	4.4(1.0)
Boys	23.3(7.0)	3.5(1.0)	43.8(6.9)	6.57(1.04)	32.9(5.3)	4.9(0.8)
Girls	34.5(7.6)	5.2(1.1)	39.8(8.7)	5.97(1.31)	25.7(5.2)	3.9(0.8)

*Note*: Minutes calculated based on 15-minute recess periods

Three separate GLMMs were conducted to determine the impact of recess frequency on observed MVPA, LPA, and SB. Covariates included temperature, organized activity, equipment, SLI.

In the MVPA model, recess frequency, *t* [40]=-3.95, *SE* = 4.54, *P* < .01; sex, *t* [40] = 4.02, *SE* = 1.82, *P* < .001, and the interaction between frequency and sex, *t* [40] = 2.31, *SE* = 3.63, *P* < .05, were significant predictors. On average across all schools, students with one daily recess engaged in significantly less MVPA per recess than students at schools with two daily recesses (*b*=-17.91, 95%CI [-27.08, -8.73]). Boys engaged in a significantly higher proportion of MVPA per recess than girls regardless of frequency (*b* = 7.29, 95%CI [3.62, 10.96]). When examined by sex, the significantly lower proportion of MVPA per recess at schools offering one daily recess persisted for both boys, *F* [40] = 4.40, *P* < .05, and girls, *F* [40] = 15.56, *P* < .001.

In the LPA model, recess frequency was a significant predictor, *t* [40] = 2.62, *SE* = 5.21, *P* < .05, while sex and the interaction between frequency and sex were not. On average across all schools, students with one daily recess engaged in significantly more LPA per recess than students at schools with two daily recesses (*b* = 13.66, 95%CI [3.12, 24.19]).

In the SB model, sex was a significant predictor, *t* [40]=-5.32, *SE* = 2.16, *P* < .001, while recess frequency and the interaction between frequency and gender were not. On average across all schools, boys engaged in a lower proportion of SB than girls per recess (*b*=-11.48, 95%CI [-15.84, -7.12]).

### Individual physical activity

A total of 142 third grade students wore accelerometers to measure individual PA during recess (ranging from 28 to 42 per school; Table 1); however, data from 134 students were included in analysis after removing those that did not wear the device for a complete recess period (n = 8). Descriptive results indicated that, on average across all four schools (six recess periods), most time at recess was spent in MVPA (*M* = 41%), followed by time in LPA (*M* = 36%) then SB (*M* = 23%). When examined by frequency, students at high frequency schools spent less time in SB (17%) compared to low frequency schools (31%), similar time in LPA (35% v. 37%), and greater time in MVPA (48%) compared to low frequency schools (33%). When converted to minutes (per 15 min), students at low frequency schools engaged in 4.9 min compared to 7.2 min for students at high frequency schools per recess. When doubled (to represent high frequency schools with two 15-minute recess periods), this equated to 14.4 MVPA minutes per day. This translates to an additional 9.5 min per day of MVPA for students attending schools with two recess periods compared to one recess. When multiplied by the average 180 days of instruction in U.S. public schools, this equates to 28 h across the school year. Full results by frequency for device-based (i.e., accelerometry) data are shown in Table 3. Full descriptive information of accelerometer data for each school are shown in Appendix 3.

**Table 3:** Mean Recess Physical Activity Levels by Recess Frequency: Accelerometry

Recess Frequency	Sedentary		Light Physical Activity		Moderate-to-Vigorous Physical Activity	
	% *M(SD)*	Minutes *M(SD)*	% (*SD*)	Minutes *M(SD)*	% *M(SD)*	Minutes *M(SD)*
Low (*n* = 2)	30.5(8.4)	4.6(1.3)	36.7(8.4)	5.5(1.3)	32.8(10.5)	4.9(1.6)
Boys	29.1(8.7)	4.4(1.3)	34.2(9.3)	5.1(1.4)	36.7(11.6)	5.5(1.7)
Girls	31.6(8.1)	4.7(1.2)	38.7(7.2)	5.8(1.1)	29.7(8.5)	4.5(1.3)
High (*n* = 2)	16.7(4.8)	2.5(0.7)	35.2(5.4)	5.3(0.8)	48.1(8.6)	7.2(1.3)
Boys	14.4(4.3)	2.2(0.7)	33.3(5.7)	5.0(0.9)	52.4(8.3)	7.9(1.2)
Girls	18.2(4.5)	2.7(0.7)	36.4(4.8)	5.5(0.7)	45.4(7.8)	6.8(1.2)

*Note*: Minutes calculated based on 15-minute recess periods

Three separate GLMMs were conducted to determine the impact of recess frequency on device-based MVPA, LPA, and SB with school income included as a covariate.

In the MVPA model, frequency, *t*(134)=-10.83, *SE* = 1.90, *P* < .001 and sex, *t*(134) = 3.90, *SE* = 1.89, *P* < .001 were significant predictors while the interaction between frequency and sex was not. On average across all schools, students with one daily recess engaged in significantly less MVPA per recess than students at schools with two daily recesses (*b*=-20.58, 95%CI [-24.34, -16.82]). Boys engaged in a significantly higher proportion of MVPA per recess than girls regardless of frequency (*b* = 7.39, 95%CI [3.65, 11.14]).

In the LPA model, frequency, *t*(134) = 6.36, *SE* = 1.18, *P* < .001, and sex, *t*(134)=-3.07, *SE* = 1.17, *P* < .01, were significant predictors while the interaction between frequency and sex was not. On average across all schools, students with one daily recess engaged in significantly more LPA per recess than students at schools with two daily recesses (*b* = 7.48, 95%CI [5.15, 9.80]). Across all recesses, boys engaged in a lower proportion of LPA than girls during recess (*b*=-3.60, 95%CI [-5.91, -1.28]).

In the SB model, frequency, *t*(134) = 8.15, *SE* = 1.61, *P* < .001, and sex, *t*(134)=-2.37, *SE* = 1.60, *P* < .05, were significant predictors while the interaction between frequency and sex was not. Students at schools with one daily recess engaged in greater proportions of SB per recess than those at schools with two or more daily recesses (*b* = 13.08, 95%CI [9.92, 16.27]). On average across all schools, boys engaged in a lower proportion of SB than girls per recess (*b*=-3.80, 95%CI [-6.96, − 0.63]).

## Discussion

The purpose of this observational study was to assess differences in children’s PA among AZ elementary schools with two or more daily recess periods, compared to those with less, in accordance with state policy ARS§ 15–118. Both group-level (observational) and individual-level (device-based) MVPA, LPA, and SB during recess were compared between schools and also between boys and girls. We found that children attending schools implementing two or more daily 15-minute recesses in accordance with ARS§ 15–118 spent an additional 5.1 (based on direct observation) and 9.5 (based on device-based accelerometry) minutes in MVPA, and 4.1 less minutes being sedentary (based on device-based accelerometry) during recess per day compared to children attending schools with one 15-minute recess period.

### Comparisons to prior studies

Our results, using both observational (group-level) and device-based (individual) data, show that children attending schools offering two or more daily recesses had greater proportions of MVPA during recess compared to schools offering less than two daily recesses. Previous studies have demonstrated that additional recess periods throughout the school day contribute to higher odds of meeting the recommended 60 min of daily PA for children [65] and improve individual levels of MVPA and overall step count [66]. One recent study of National Youth Fitness Surveillance data showed that children with the highest level of parent-reported recess provision (e.g., 30 or more daily recess minutes), had two-fold higher odds of meeting national standards of 60 min daily of MVPA measured using accelerometry, compared to children with the lowest recess provision [65]. In our study, children’s MVPA at high-frequency schools was greater during both recess periods compared to MVPA levels at low-frequency schools, countering the idea that children compensate by engaging in higher levels of PA when not offered additional opportunities to be active. Similar research has been reported in the physical education literature where children’s PA levels are significantly higher on physical education days than non-physical education days [67–70], suggesting that greater levels of PA during physical education and recess may be indicative of habitual PA among children [69] and higher recess frequency may be an indicator of an overall supportive culture of PA within a school, rather than contributing to a compensatory PA effect.

Our device-based (individual-level) results indicate that children attending schools with two or more daily recesses spend less time sedentary compared to schools with one daily recess, an important finding considering that sedentary time among children at recess is associated with poor fitness [71]. Children who spend more than 15 min of recess time being sedentary are more than 40 times more likely to have unhealthy levels of cardiorespiratory fitness compared to children who spend less than 15 recess minutes sedentary [72]. Results of both device-based and observational data sources indicated that children at schools with one recess engaged in more light PA compared to those at schools with two recess periods. Although these differences were slight (average time in light PA was less than one minute different between schools with high and low recess frequency), light PA is an important health behavior and associated with reduced disease risk including decreased inflammation [73] and excess adiposity [74] among children. Also, while not a focus of our study, increasing opportunities for PA through increased recess frequency may also help improve physical and social health outcomes [30]. Our findings that boys engaged in significantly more MVPA and less SB than girls is consistent with literature reviews reporting that boys are more physically active compared to girls at recess [75].

### Individual vs. group physical activity

Direct observation and accelerometry, both considered forms of objective PA measurement, were used concurrently in the present study. Direct observation is regarded as the gold standard method for assessing PA at recess because it involves in-person observation of behavior [76] and allows for the assessment of environmental support factors during recess (e.g., presence of equipment, organized activity) that can often only be assessed with physical presence [76]. Accelerometry is a commonly used measure for school-aged children and allows for accurate, device-based, and continuous monitoring of PA at the individual level [77]. Results from direct observation and accelerometry often do not agree with each other [78]. In our study, individual proportions of activity levels during recess between direct observation and accelerometry differed (e.g., overall MVPA from direct observation = 28% v. accelerometry = 41%), resulting in some variations between the estimated number of minutes spent in each activity level during a 15-minute recess period. To enhance wear compliance, the accelerometers were positioned on the wrist. Studies have shown that wrist-worn accelerometers can lead to an overestimation of MVPA [79]. This overestimation is attributed to the devices capturing arm movements, which may not accurately reflect whole-body physical activity [80]. Furthermore, the dynamic and sporadic movements of children’s activities may be inaccurately classified as MVPA. The device’s challenge in distinguishing between various movement types contributes to an overall exaggeration of PA intensity [81]. Consequently, this accelerometry-induced overestimation may contribute to observed disparities between direct observation and accelerometer-derived data.

However, the overall trend of our results from the regression analyses were similar between direct observation and accelerometry, due to the following possible reasons: (1) the duration of the recess periods were relatively short, (2) the trained observers coded the activity intensity accurately, (3) compliance with accelerometer wearing was high, and (4) the accelerometer data processing was appropriate.

### Policy implications

Supporting the larger project’s adapted ecological model of PA policy, the current study showed the importance of state policy, school environment (e.g., environmental support), and individual factors (e.g., student demographics, such as gender) that support student physical activity and health at school [41]. The number of states with laws mandating recess in schools has increased over the past decade in the United States, with 12 states currently mandating recess explicitly and eight states with active proposed legislation in support of recess as confirmed by all state government websites reporting on school PA laws at the time of publication in 2023. As with school-based PA laws, however, the lone existence of a policy does not equate to adherence [19, 33] and current recess legislation and practices vary widely [38]. For example, school-based PA policies that are more comprehensive and include specific language (e.g., prohibit withholding) are positively related to children’s PA at recess [41].

Our results support the positive health impact of considering frequency in recess policy, and recommending at least 30 min of recess per day. In the case of this research in Arizona, policy-compliant schools offered 30-minutes of daily recess, which may be a good target for other states. Current US national recommendations for schools to implement 20 min of recess [82, 83] may be overly restrictive, considering the literature base supporting an array of benefits of more recess [83, 84], including children’s overall PA and body mass index [66, 85, 86]. However, future studies should investigate different recess durations to determine optimum length for health gains while also considering preferences of school staff and competing demands from other academic classes and content areas during the school day.

This study is not without limitations. Although our sample was randomly selected, included a diverse range of income levels, and was focused on schools in a large county (over 60% of the AZ population resides in Maricopa County); [87] the sample size of four schools was small and may not be representative of all schools, particularly those in rural areas. Additionally, although our sample size of students in grade three is similar to schools in Maricopa County, Arizona generally has higher average school enrollment and diverse racial and ethnic populations compared to other elementary schools in the United States [59]. Our use of both observations and device-based assessment provided a more comprehensive battery of measures compared to one technique alone; however, comparisons should be made with caution as observational data included average activity levels among all students across multiple recess observations whereas device data included averages among individuals across all eligible recess periods. During systematic observations, researchers made judgements about child sex based on visual characteristics. Although this aligns with current practice, we note that this strategy may be biased as researchers were not aware of the actual sex of children observed in the study and made assumptions based on gendered perceptions. We also note that our study was cross-sectional so causal conclusions cannot be assumed.

## Conclusions

Schools are ideal spaces for targeted behavior change and population health improvement due to their accessible systems of support and service for children. Our study supports the positive impact of two daily recess periods on children’s moderate-to-vigorous physical activity and sedentary behavior. Adding an additional recess period to the school day may contribute up to 28 additional hours of MVPA for children through-out a 180-day school year. Considering language that specifies frequency, and potentially duration, in state-level policies can support widespread benefits for children’s health.

### Electronic supplementary material

Below is the link to the electronic supplementary material.

Supplementary Material 1

## Data Availability

The datasets supporting the conclusions of this article are available in the ASU Library Research Data Repository [10.48349/ASU/D4FR67].

## Abbreviations

AZ: Arizona
PA: Physical activity
MVPA: Moderate to vigorous physical activity
LPA: Light physical activity
SB: Sedentary behavior
SOPLAY: System for Observing Play and Leisure Activity in Youth
GLMM: General linear mixed effects models
