# Correction to: A natural experiment to assess recess frequency on children’s physical activity in Arizona (U.S.) elementary schools

**DOI:** 10.1186/s12889-024-19271-6

**Published:** 2024-07-08

**Authors:** Allison Poulos, Kylie Wilson, Marissa Schulke, Kahyun Nam, Punam Ohri-Vachaspati, Yang Bai, Pamela Hodges Kulinna

**Affiliations:** 1 College of Health Solutions, Arizona State University, ABC 222 425 North 5th Street, Phoenix, AZ 85004 USA; 2 Mary Lou Fulton Teachers College, Arizona State University, Tempe, AZ 85281 USA; 3 Department of Health and Kinesiology, University of Utah, Salt Lake City, UT 84112 USA

**Correction to**: ***BMC Public Health*** **24, 225 (2024)**

10.1186/s12889-023-17605-4

The original publication of this article contained 2 errors in the result section and 1 error in the conclusion section. The incorrect and correct information is listed in this correction article. This does not affect the overall conclusion, the original article has been updated.

**Incorrect**

**Results**

- This equates to 8.8 MVPA minutes per day, or an additional 5.1 min per day of MVPA among students attending schools offering two recess periods compared to one. Full results by recess frequency for observational data are shown in Table 2.
- This translates to an additional 9.5 min per day of MVPA for students attending schools with two recess periods compared to one recess. Full results by frequency for device-based (i.e., accelerometry) data are shown in Table 3. Full descriptive information of accelerometer data for each school are shown in Appendix 3.

**Conclusion**

- Adding an additional recess period to the school day **may 15 h of** MVPA for children throughout the school year. Considering language that specifies frequency, and potentially duration, in state-level policies can support widespread benefits for children’s health.

**Correct**

**Results**

- This equates to 8.8 MVPA minutes per day, or an additional 5.1 min per day of MVPA among students attending schools offering two recess periods compared to one. **When multiplied by the average 180 days of instruction in U.S. public schools, this equates to 15 h across the school year.** Full results by recess frequency for observational data are shown in Table 2.
- This translates to an additional 9.5 min per day of MVPA for students attending schools with two recess periods compared to one recess. **When multiplied by the average 180 days of instruction in U.S. public schools, this equates to 28 h across the school year**. Full results by frequency for device-based (i.e., accelerometry) data are shown in Table 3. Full descriptive information of accelerometer data for each school are shown in Appendix 3.

**Conclusion**

- Adding an additional recess period to the school day **may contribute up to 28 additional hours of** MVPA for children through-out **a 180-day** school year. Considering language that specifies frequency, and potentially duration, in state-level policies can support widespread benefits for children’s health.

