# Supplementary material for: A natural experiment to assess recess frequency on children’s physical activity in Arizona (U.S.) elementary schools
Source: BMC Public Health. 2024 Jan 18;24:225. doi: 10.1186/s12889-023-17605-4 (PMC10797942; doi:10.1186/s12889-023-17605-4)
Supplement: Supplementary file 1 — Supplementary Material 1 [file 12889_2023_17605_MOESM1_ESM.docx]

Appendix

Appendix 1. Adapted ecological model of physical activity policy to assess the stages of implementation and dissemination of ARS 15-118 affecting children’s physical activity


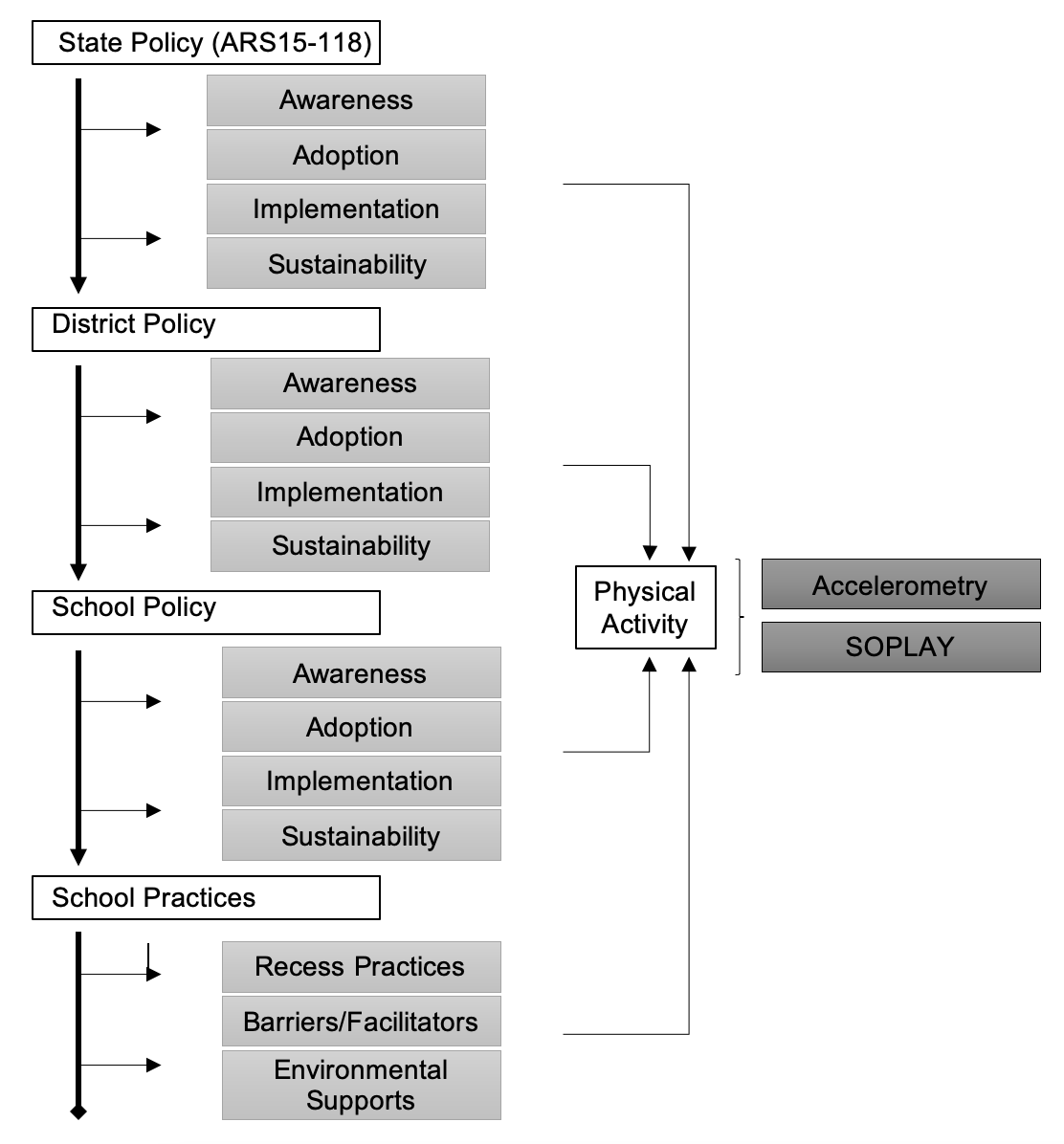


Appendix 2: Mean Recess Physical Activity Levels by Sex and School: Direct Observation

|  |  | Sedentary | | | Light Physical Activity | | | Moderate-to-Vigorous Physical Activity | | |
| --- | --- | --- | --- | --- | --- | --- | --- | --- | --- | --- |
|  |  | %(*SD*) | Minutes(*SD)* | | %(*SD*) | Minutes(*SD)* | | %(*SD*) | Minutes(*SD)* | |
| Overall | | 29.3(9.2) | 4.4 | (1.4) | 43.1(7.2) | 6.5 | (1.1) | 27.7(7.7) | 4.2 | (1.16) |
|  | Boys | 23.2(6.1) | 3.5 | (0.9) | 44.4(6.2) | 6.7 | (0.9) | 32.4(4.9) | 4.87 | 9(0.74) |
|  | Girls | 35.4(7.7) | 5.3 | (1.2) | 41.7(8.0) | 6.3 | (1.2) | 22.9(7.2) | 3.44 | (1.07) |
| School | |  |  |  |  |  |  |  |  |  |
|  | School LF1 | 27.6(9.9) | 4.1 | (1.5) | 46.1(4.6) | 6.9 | (0.7) | 26.3(9.5) | 4.0 | (1.4) |
|  | Boys | 21.0(3.6) | 3.2 | (0.5) | 45.9(5.5) | 6.9 | (0.8) | 33.2(4.2) | 5.0 | (0.6) |
|  | Girls | 34.1(9.9) | 5.1 | (1.5) | 46.4(4.5) | 7.0 | (0.7) | 19.5(8.2) | 2.9 | (1.2) |
|  | School LF2 | 33.4(9.2) | 5.0 | (1.4) | 45.1(3.4) | 6.8 | (0.5) | 21.6(9.2) | 3.2 | (1.4) |
|  | Boys | 25.4(3.5) | 3.8 | (0.5) | 45.4(3.6) | 6.8 | (0.5) | 29.3(4.0) | 4.4 | (0.6) |
|  | Girls | 41.3(2.8) | 6.2 | (0.4) | 44.8(4.1) | 6.7 | (0.6) | 13.9(6.8) | 2.1 | (1.0) |
|  | School HF1 | 31.8(9.2) | 4.8 | (1.3) | 39.5(8.9) | 5.9 | (1.3) | 28.7(6.7) | 4.3 | (1.0) |
|  | Boys | 26.0(5.3) | 3.9 | (0.8) | 41.7(7.4) | 6.3 | (1.1) | 32.3(5.1) | 4.8 | (0.8) |
|  | Girls | 37.6(8.7) | 5.6 | (1.3) | 37.2(10.2) | 5.6 | (1.5) | 25.2(6.4) | 3.8 | (1.0) |
|  | School HF2 | 26.4(8.6) | 4.0 | (1.3) | 43.9(6.8) | 6.6 | (1.0) | 29.7(6.1) | 4.5 | (0.9) |
|  | Boys | 20.9(7.7) | 3.1 | (1.2) | 45.7(6.5) | 6.9 | (1.0) | 33.4(5.7) | 5.0 | (0.9) |
|  | Girls | 31.8(5.8) | 4.8 | (0.9) | 42.1(7.1) | 6.3 | (1.1) | 26.1(4.2) | 3.9 | (0.6) |

*Note*: Minutes calculated based on 15-minute recess periods. LF=Low frequency (<2 recesses). HF=High frequency (2+ recesses).

Appendix 3: Mean Recess Physical Activity Levels by Sex and School: Accelerometry

|  |  | Sedentary | | |  | Light Physical Activity | | |  | Moderate-to-Vigorous Physical Activity | | |  |
| --- | --- | --- | --- | --- | --- | --- | --- | --- | --- | --- | --- | --- | --- |
|  |  | % | (*SD*) | Minutes | (*SD*) | % | (*SD*) | Minutes | (*SD*) | % | (*SD*) | Minutes | (*SD*) |
| Overall (*n*=4) | | 23. | 3(9.7) | 3.5 | (1.5) | 35.9 | (7.0) | 5.4 | (1.1) | 40.8 | (12.2) | 6.1 | (1.8) |
|  | Boys | 22.0 | (10.1) | 3.3 | (1.5) | 33.7 | (7.2) | 5.1 | (1.1) | 44.2 | (12.8) | 6.6 | (1.9) |
|  | Girls | 24.2 | (9.3) | 3.6 | (1.4) | 37.5 | (6.1) | 5.6 | (0.9) | 38.4 | (11.3) | 5.8 | (1.7) |
| School | |  |  |  |  |  |  |  |  |  |  |  |  |
|  | School LF1 | 30.4 | (10.4) | 4.6 | (1.6) | 43.2 | (4.3) | 6.5 | (0.7) | 26.4 | (8.1) | 4.0 | (1.2) |
|  | Boys | 28.8 | (11.5) | 4.3 | (1.7) | 43.3 | (3.8) | 6.5 | (0.6) | 28.0 | (9.0) | 4.2 | (1.4) |
|  | Girls | 31.4 | (9.9) | 4.7 | (1.5) | 43.2 | (4.7) | 6.5 | (0.7) | 25.5 | (7.5) | 3.8 | (1.1) |
|  | School LF2 | 30.6 | (5.2) | 4.6 | (0.8) | 28.8 | (4.5) | 4.3 | (0.7) | 40.6 | (7.4) | 6.1 | (1.1) |
|  | Boys | 29.4 | (5.8) | 4.4 | (0.9) | 26.9 | (4.6) | 4.0 | (0.7) | 43.7 | (8.1) | 6.6 | (1.2) |
|  | Girls | 32.0 | (4.2) | 4.8 | (0.6) | 31.2 | (3.0) | 4.7 | (0.5) | 36.8 | (4.3) | 5.5 | (0.7) |
|  | School HF1 | 15.6 | (4.8) | 2.4 | (0.7) | 34.9 | (5.8) | 5.2 | (0.9) | 49.5 | (9.1) | 7.4 | (1.4) |
|  | Boys | 13.8 | (4.7) | 2.1 | (0.7) | 33.6 | (6.5) | 5.0 | (1.0) | 52.6 | (9.4) | 7.9 | (1.4) |
|  | Girls | 17.3 | (4.4) | 2.6 | (0.7) | 36.1 | (5.0) | 5.4 | (0.8) | 46.6 | (7.9) | 7.0 | (1.2) |
|  | School HF2 | 18.3 | (4.4) | 2.8 | (0.7) | 35.6 | (4.7) | 5.3 | (0.7) | 46.1 | (7.6) | 6.9 | (1.2) |
|  | Boys | 16.0 | (2.7) | 2.4 | (0.4) | 32.4 | (2.8) | 4.9 | (0.4) | 51.6 | (4.3) | 7.7 | (0.6) |
|  | Girls | 19.0 | (4.6) | 2.9 | (0.7) | 36.7 | (4.8) | 5.5 | (0.7) | 44.2 | (7.7) | 6.6 | (1.2) |

*Note*: Minutes calculated based on 15-minute recess periods. LF=Low frequency (<2 recesses). HF=High frequency (2+ recesses).
